# Genetic diversity of gliadin-coding alleles in bread wheat (*Triticum aestivum* L.) from Northern Kazakhstan

**DOI:** 10.7717/peerj.7082

**Published:** 2019-06-12

**Authors:** Maral Utebayev, Svetlana Dashkevich, Nina Bome, Kulpash Bulatova, Yuri Shavrukov

**Affiliations:** 1 A.I. Barayev Research and Production Centre of Grain Farming, Shortandy, Kazakhstan; 2 Institute of Biology, University of Tyumen, Tyumen, Russia; 3 Kazakh Research Institute of Agriculture and Plant Growing, Almaty region, Kazakhstan; 4 College of Science and Engineering, School of Biological Sciences, Flinders University, Bedford Park, SA, Australia

**Keywords:** Allele frequency, Protein electrophoresis, Genetic polymorphism, Gli loci, Gliadins, Bread wheat

## Abstract

**Background:**

Spring bread wheat (*Triticum aestivum* L.) represents the main cereal crop in Northern Kazakhstan. The quality of wheat grain and flour strongly depends on the structure of gluten, comprised of gliadin and glutenin proteins. Electrophoresis spectra of gliadins are not altered by environmental conditions or plant growth, are easily reproducible and very useful for wheat germplasm identification in addition to DNA markers. Genetic polymorphism of two *Gli* loci encoding gliadins can be used for selection of preferable genotypes of wheat with high grain quality.

**Methods:**

Polyacrylamide gel electrophoresis was used to analyse genetic diversity of gliadins in a germplasm collection of spring bread wheat from Northern Kazakhstan.

**Results:**

The highest frequencies of gliadin alleles were found as follows, in *Gli1*: *-A1****f*** (39.3%), *-B1****e*** (71.9%), and *-D1****a*** (41.0%); and in *Gli-2*: *-A2****q*** (17.8%), *-B2****t*** (13.5%), and *-D2****q*** (20.4%). The combination of these alleles in a single genotype may be associated with higher quality of grain as well as better adaptation to the dry environment of Northern Kazakhstan; preferable for wheat breeding in locations with similar conditions.

## Introduction

Wheat flour remains one of main ingredients in quite a diverse range of foods for human consumption and provides the major proteins gliadins and glutenins. In particular, glutenin can make up at least 40% of the total protein in grain and flour (Qi et al., 2006; Metakovsky et al., 2018). The genetic control of gliadin includes two major genes, *Gli-1* and *Gli-2*, mapped to the short arms of chromosome groups 1 and 6, respectively, with corresponding homeologous genes, *Gli-A1*, *-B1*, *-D1* and *Gli-A2*, *-B2*, *-D2* (Metakovsky, Branlard & Graybosch, 2006; Metakovsky et al., 2018). Multiple alleles are typically found for both *Gli* loci. Each *Gli* allele encodes the transcription of clusters of subunits, with several components of gliadin proteins showing linked inheritance. Gliadin groups can differ in the number of components, their electrophoretic mobility and molecular weight, and levels of expression (Sozinov & Poperelya, 1980; Obukhova & Shumny, 2016). By its nature, gliadin is a complex protein with several components that can be separated using polyacrylamide gel electrophoresis in aluminium-lactate buffer (pH = 3.1) (Bushuk & Zillman, 1978). The original protocol of gliadin electrophoresis has been since modified (Tkachuk & Metlish, 1980; Khan, Hamada & Patek, 1985; Metakovsky & Novoselskaya, 1991), and was used as the basis for the International standard procedure ISO (ISO, 1993). *Gli* alleles and their components have been widely studied and identified in International wheat germplasm collections, resulting in published Catalogues. The genetic polymorphism in the composition of *Gli* alleles in a given genotype was summarised as the ‘Gliadin genetic formula’ (GGF) in the Catalogues for bread wheat (Metakovsky, 1991; Metakovsky et al., 2018) and for durum wheat (Melnikova, Kudryavtseva & Kudryavtsev, 2012).

As reported in many publications, wheat cultivars produced in each separate country often have similar GGF despite the absence of any selection pressure based on gliadins (Xynias, Kozub & Sozinov, 2006; Aguiriano et al., 2008; Salavati et al., 2008; Melnikova et al., 2010; Novoselskaya-Dragovich et al., 2011; Hailegiorgis, Lee & Yun, 2017). A linkage between *Gli* alleles and other genes or a group of genes encoding favourable traits can be preferable and beneficial for wheat breeding (Chebotar et al., 2012). Therefore, a high frequency of *Gli* alleles can be used as simple and convenient method based on protein marker analysis for wheat germplasm identification and application in further breeding programs in the same environment.

Currently, molecular markers based on DNA analysis are widely used for genotyping and genetic identification in various crops (Shavrukov, 2016; Jatayev et al., 2017; Scheben, Batley & Edwards, 2017; Burridge et al., 2018). The application of molecular markers was successful in the study of wheat genes controlling such traits as 1,000-grain weight, protein and gluten content (Zhang et al., 2018), grain hardness (Nirmal et al., 2016), flour production from grain milling (Nirmal et al., 2017), and bread quality (Henry, Furtado & Rangan, 2018). Genome editing using CRISPR/Cas9 technology represents a novel method in plants (Khlestkina & Shumny, 2016; Liang et al., 2018; Borisjuk et al., 2019), for production of wheat with low gluten content (Sánchez-León et al., 2018), as required by people allergic to some components of gliadin in traditional wheat cultivars (Palosuo et al., 2001; Pastorello et al., 2007).

Nevertheless, molecular markers are relatively expensive in the equipment and reagents required, in typically well-established molecular laboratories. In contrast, biochemical markers based on proteins such as enzymes and storage proteins offer an alternative method involving cheaper and simpler protocols for crop breeding including wheat (Shewry & Halford, 2001; Ghanti et al., 2009; Al-Doss et al., 2010; Netsvetaev, Akinshina & Bondarenko, 2010; Hailegiorgis, Lee & Yun, 2017). Additionally, protein synthesis is encoded by genes, and modulation of gene expression in response to changes in the environment directly results in different levels of the corresponding proteins.

The aim of this study was to identify and analyse the genetic diversity of the *Gli* alleles in spring bread wheat (*Triticum aestivum* L.) collection from Northern Kazakhstan, and to address the question of which alleles of gliadins with highest frequencies are typical for modern wheat produced and cultivated in the dry environment of this region.

## Materials and Methods

### Wheat germplasm and geographic locations

**Figure 1:**
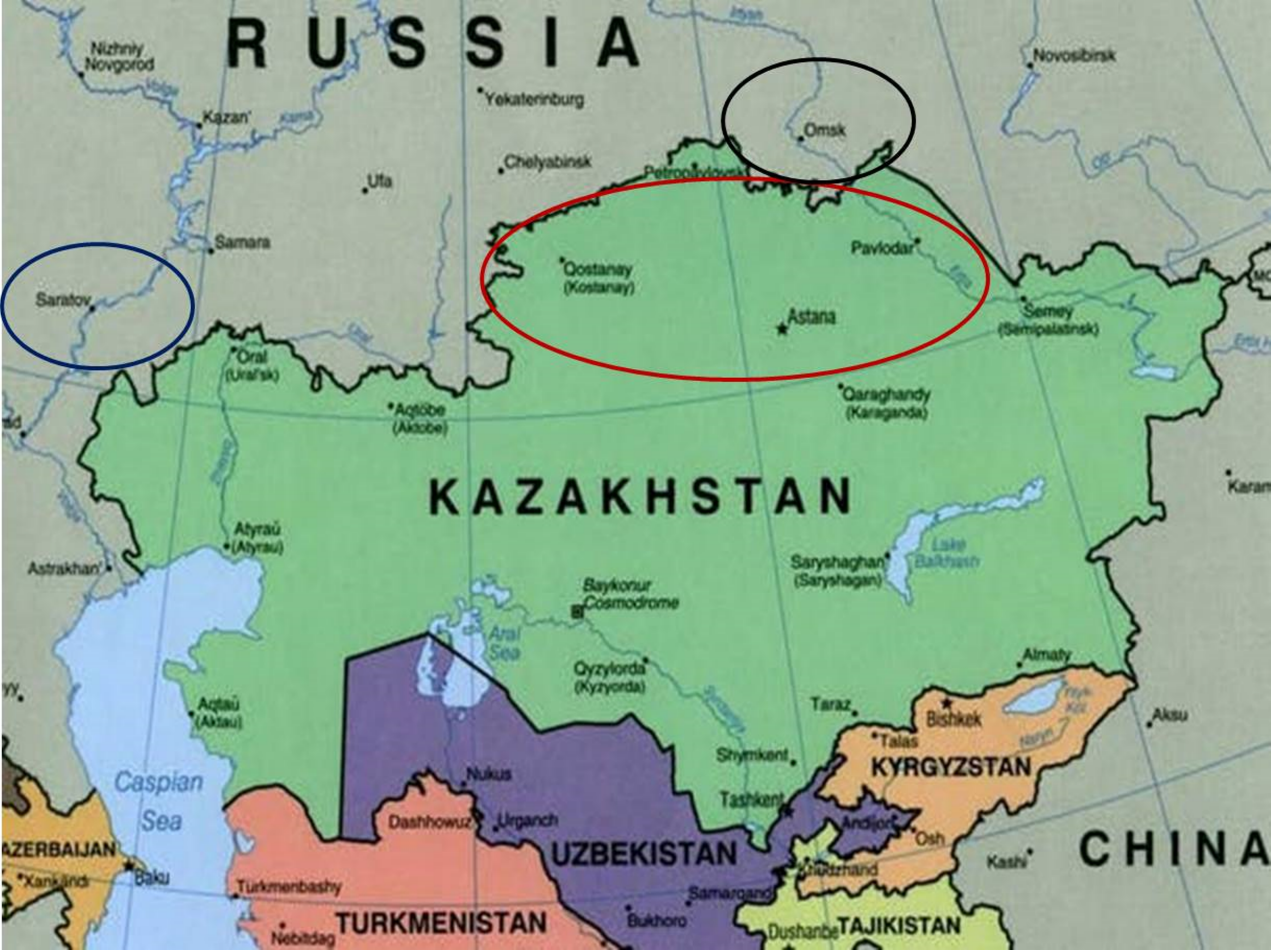
A map of Kazakhstan and nearby regions of Russia. The red oval shows Northern Kazakhstan, while the Russian regions, Saratov (European part) and Omsk (Siberia), are shown in blue and black, respectively (Kusznir, 2015). The map was taken from the website: http://theconversation.com/russias-borders-moscows-long-alliance-with-kazakhstan-is-strong-but-not-unbreakable-36457.

A seed collection of 70 bread wheat cultivars was provided by the A.I. Barayev Research and Production Centre of Grain Farming, Shortandy, Kazakhstan. The studied wheat accessions were bred and produced at different times by Breeding Institutions (Karabalyk Agricultural Breeding Station and Pavlodar Research Institute of Agriculture) in Northern Kazakhstan, as listed in Supplemental Information 1. Additional data for various wheat germplasms from Kazakhstan and neighbouring regions, used for comparison of the results obtained for genetic diversity of *Gli* alleles in wheats, were retrieved from papers published earlier (Supplemental Information 2). In the map (Fig. 1), Northern Kazakhstan and two nearby regions in Russia with wheat Breeding Research Organisations—Saratov (European part) and Omsk (Siberia) are indicated by ovals. Briefly, Northern Kazakhstan is located at latitude 51°–55°N and longitude 61°–79°E, with a territory of about 565K km^2^ comprised largely of steppe or low-hilled forest. The strong continental climate is characterized by a cold and long winter with high winds, but a hot and short summer season. Average winter/summer temperatures are about −18 °C and 20 °C but extreme levels of −45 °C and 41 °C, respectively can also be reached.

### Electrophoresis and identification of *Gli* alleles

Polyacrylamide gel electrophoresis was carried out following a method published earlier (Metakovsky & Novoselskaya, 1991). Gliadins were extracted from individually milled seeds by adding 150 µL of 70% ethanol. Acrylamide polymerization was initiated by 50 µL of 3% H_2_O_2_ in 45 mL of gel solution. A vertical gel tank, model VE-20 (Helicon, Moskva, Russia) was used and the gels were 17.8 × 17.5 × 1 (mm) in size. Electrophoresis was conducted at optimal temperature below 20 °C, at 520 V for 4 h. 10% trichloroacetic acid supplied with 0.05% of Coomassie Brilliant Blue R-250 in ethanol (Sigma-Aldrich, St. Louis, MO, USA) was used for gel fixation and staining. The identification of gliadin components was conducted using the Protein Catalogue (Metakovsky, 1991). Genes that encoded gliadins were identified in accordance to the Gene Catalogue developed by McIntosh et al. (2008) for *Gli-1* (*-A1*, *-B1*, and *-D1*) and for *Gli-2* (*-A2*, *-B2*, and *-D2*). Alleles of the *Gli* locus were designated as additional Latin letters and total GGFs were used as recommended for wheat cv. Chinese Spring with the following in full GGF: *Gli-A1**a***, *Gli-B1**a***, *Gli-D1**a***, *Gli-A2**a***, *Gli-B2**a***, *Gli-D2**a***; and abbreviated GGF: ***a** , **a** , **a** , **a** , **a** , **a***.

### Computer and statistical analysis

Intra-population diversity (*μ* ± *S*_*μ*_) and frequency of rare alleles (*h* ± *S*_*h*_) were calculated following the method published by Zhivotovsky (1991), while genetic diversity (*H*) was calculated by the formula described by Nei, where *p*_*i*_ is the frequency of alleles (Nei, 1973)

\begin{eqnarray*}\mathbi{H}=1-\sum \mathbi{p}_{\mathbi{i}}^{2}. \end{eqnarray*}

Phylogenetic tree construction and clustering analysis among the studied wheat genotypes was carried out using the computer program software Statistica 6.0. (Statsoft, USA) following instructions for Ward’s method with Manhattan distances and applied for GGFs. The ‘Data Standardization’ option was applied to transform the allele identifications in letters into numbers suitable for the computer program software (http://documentation.statsoft.com/STATISTICAHelp.aspx?path=Cluster/ClusterAnalysis/Examples/Example1JoiningTreeClustering).

## Results

### *Gli* allele diversity

**Figure 2:**
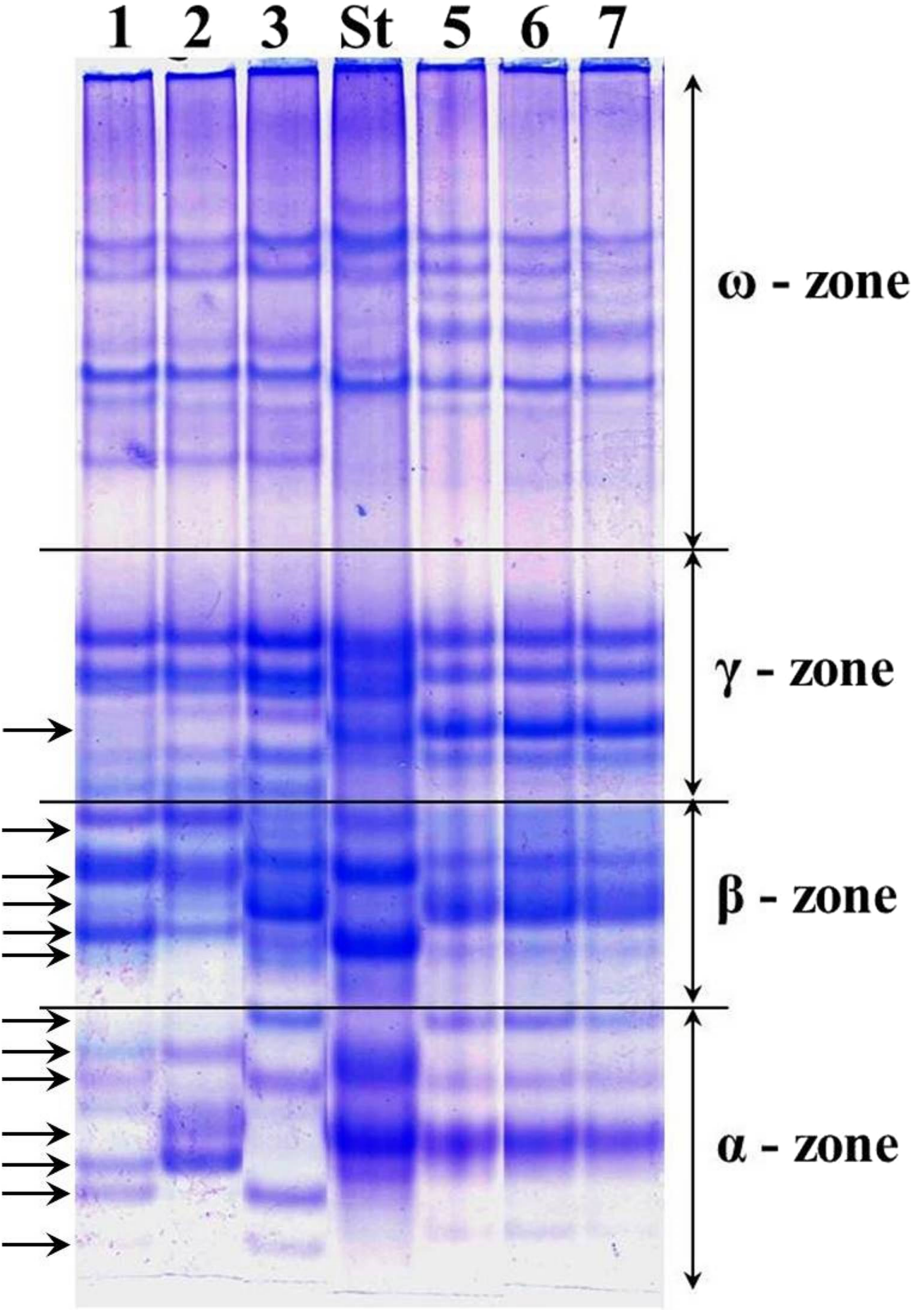
Electrophoregram of the gliadin spectrum of polymorphic cv. Lutescence 65 (Lanes 1–3) in comparison to cv. Bezostaya 1 (Lane 4, used as a Standard) and monomorphic cv. Byrlestik (Lanes 5–7). Subfractions *α*, *β*, *γ* and *ω* with polymorphic bands are indicated.

The alleles of loci *Gli-1* and *Gli-2* identified in the wheat germplasm collection (70 accessions) and their GGFs are presented in Supplemental Information 1. Most of the studied wheats were monomorphic (76%) while the remaining 24% accessions were polymorphic. Grains of such polymorphic wheats consisted of a mixture of genotypes, with variable alleles in one or more *Gli* loci. For example, several biotypes of gliadins were present in polymorphic cv. Lutescence 65 with various spectra of gliadin components in three zones, *α*-, *β*- and *γ*, but identical in *ω*-zone (Fig. 2, lanes 1–3). Plants of cv. Byrlestik were monomorphic and represent the single type of gliadin spectrum (Fig. 2, lanes 5–7). In general, the inter-cultivar polymorphic alleles of *Gli* encode the biosynthesis of gliadin components located in all four zones (*α*-, *β*-, *γ*- and *ω*-zones) of the gliadin spectrum on the polyacrylamide gel electrophoregram (Fig. 2).

**Figure 3:**
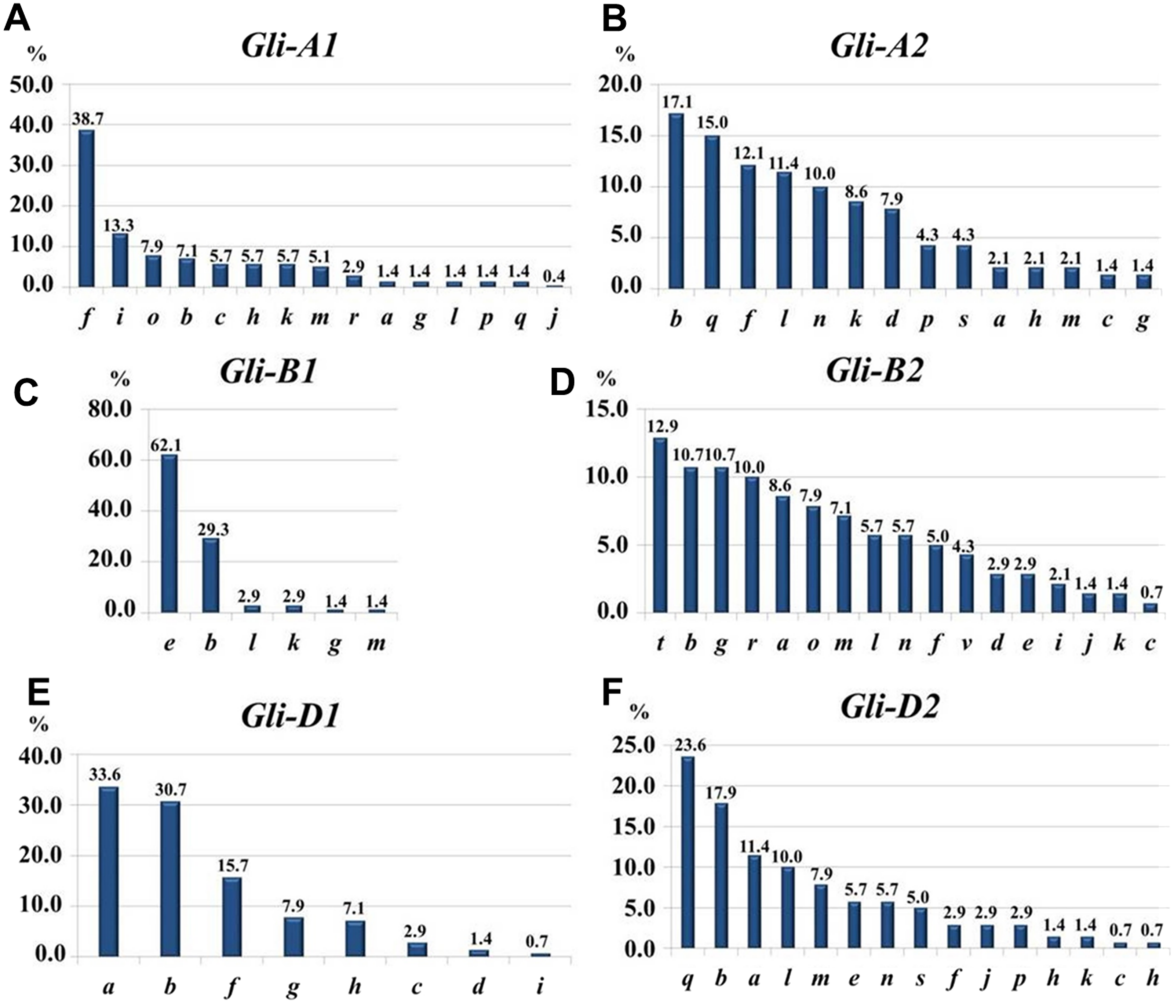
Allele frequencies in *Gli* loci identified in the studied collection of 70 accessions of spring bread wheat from Northern Kazakhstan. .

At the *Gli-1* locus, the highest frequencies were found in alleles *Gli-A1**f*** (38.7%), *-B1****e*** (62.1%), and *-D1**a*** (33.6%). In contrast, the level of highest frequency of alleles was smaller at the *Gli-2* locus and comprising *Gli-A2**b*** (17.14%), *-B2**t*** (12.9%), and *-D2**q*** (23.6%). Therefore, the GGF of the majority of wheats bred and cultivated in Northern Kazakhstan is: ***f, e, a, b, t, q,*** based on highest frequencies of the alleles. In total, results of gliadin electrophoresis revealed six and eight alleles in *Gli-B1* and *Gli-D1* loci, respectively, 14 alleles in each of three loci, *Gli-A1*, *Gli-A2* and *Gli-D2*, and 17 alleles in *Gli-B2* locus (Fig. 3).

Levels of genetic diversity (*H*), intra-population diversity (*μ*) and frequencies of rare alleles (*h*) in 70 wheat accessions from Northern Kazakhstan were calculated based on allele frequencies in *Gli* loci from Supplemental Information 1 and are presented in Table 1A. For comparison, four other studies of wheat from Northern Kazakhstan (Supplemental Information 2) with partial overlap in the accessions studied were joined together with the current study, with the combined results for 139 wheat accessions in total from Northern Kazakhstan presented in Table 1B.

**Table 1:** Genetic diversity (*H*), intra-population diversity (*μ*) and frequencies of rare alleles (*h*) in 70 (A) and 139 combined (B) wheat accessions from Northern Kazakhstan.

Diversity estimates	Gliadin-coding *Gli* loci					
	*A1*	*B1*	*D1*	*A2*	*B2*	*D2*
**A.** 70 wheat accessions from Northern Kazakhstan (Supplemental Information 1)						
*H*	0.81	0.53	0.76	0.89	0.92	0.87
*μ* ± *S*_*μ*_	10.43 ± 0.73	3.65 ± 0.35	6.00 ± 0.41	12.04 ± 0.58	15.13 ± 0.64	11.56 ± 0.63
*h* ± *S*_*h*_	0.25 ± 0.05	0.39 ± 0.06	0.25 ± 0.05	0.14 ± 0.04	0.11 ± 0.04	0.17 ± 0.05
**B.** 139 wheat accessions from Northern Kazakhstan (Supplemental Informations 1 and 2)						
*H*	0.80	0.45	0.75	0.90	0.93	0.89
*μ* ± *S*_*μ*_	12.32 ± 0.71	4.33 ± 0.18	6.78 ± 0.40	14.44 ± 0.61	17.37 ± 0.57	13.88 ± 0.56
*h* ± *S*_*h*_	0.32 ± 0.04	0.52 ± 0.04	0.32 ± 0.04	0.20 ± 0.03	0.13 ± 0.03	0.18 ± 0.03

Most of the results presented in Tables 1A and 1B are very similar, indicating for a representable subset of 70 wheat accessions for Northern Kazakhstan. For example, genetic diversity, *H*, was highest in loci *Gli-B2* (0.92/0.93) and *Gli-A2* (0.89/0.90), while smallest *H* = 0.53/0.45 were calculated for *Gli-B1* in both parts of Table 1. The same trend has been found for intra-population diversity *μ* = 15.13∕17.37 and 12.04/14.44 for alleles of loci *Gli-B2* and *Gli-A2*, respectively, with maximal number of the identified alleles (17 and 14 alleles, respectively). In contrast, the locus *Gli-B1* had the smallest value of *μ* = 3.65∕4.33 with only six identified alleles as the smallest number in this study and with highest frequency of the *Gli-B1**e*** allele (Table 1, Fig. 3).

The structure of intra-population diversity can be characterised by the frequencies of rare alleles (*h*). A population can be estimated as ‘balanced’ if values of *h* are less than 0.3 and as small as possible (Zhivotovsky, 1980). Therefore, the most balanced for intra-population diversity was found for locus *Gli-B2* (*h* = 0.11/0.13), while locus *Gli-B1* had the highest value for *h* (0.39/0.52) due to the highest frequency of a single allele, *Gli-B1**e***.

The highest frequencies of each gliadin allele in the combined group of 139 wheat accessions were accounted as: *Gli-A1**f*** (39.3%), *-B1**e*** (71.9%), *-D1**a*** (41.0%), *-A2**q*** (17.8%), *-B2**t*** (13.5%), and *-D2**q*** (20.4%). The GGF in the analysis of 139 wheat accession was as follows: ***f, e, a, q, t, q***, and almost identical to those identified in the current study, with only a single difference for *Gli-A2-* ***q*** or -***b***. Therefore, the most typical GGF in wheat accessions from Northern Kazakhstan can be identified as: ***f, e, a, q*** + ***b, t, q***.

### Comparative phylogenetic analysis of the biodiversity of gliadin- coding loci in bread wheat from Northern Kazakhstan and other origins

**Figure 4:**
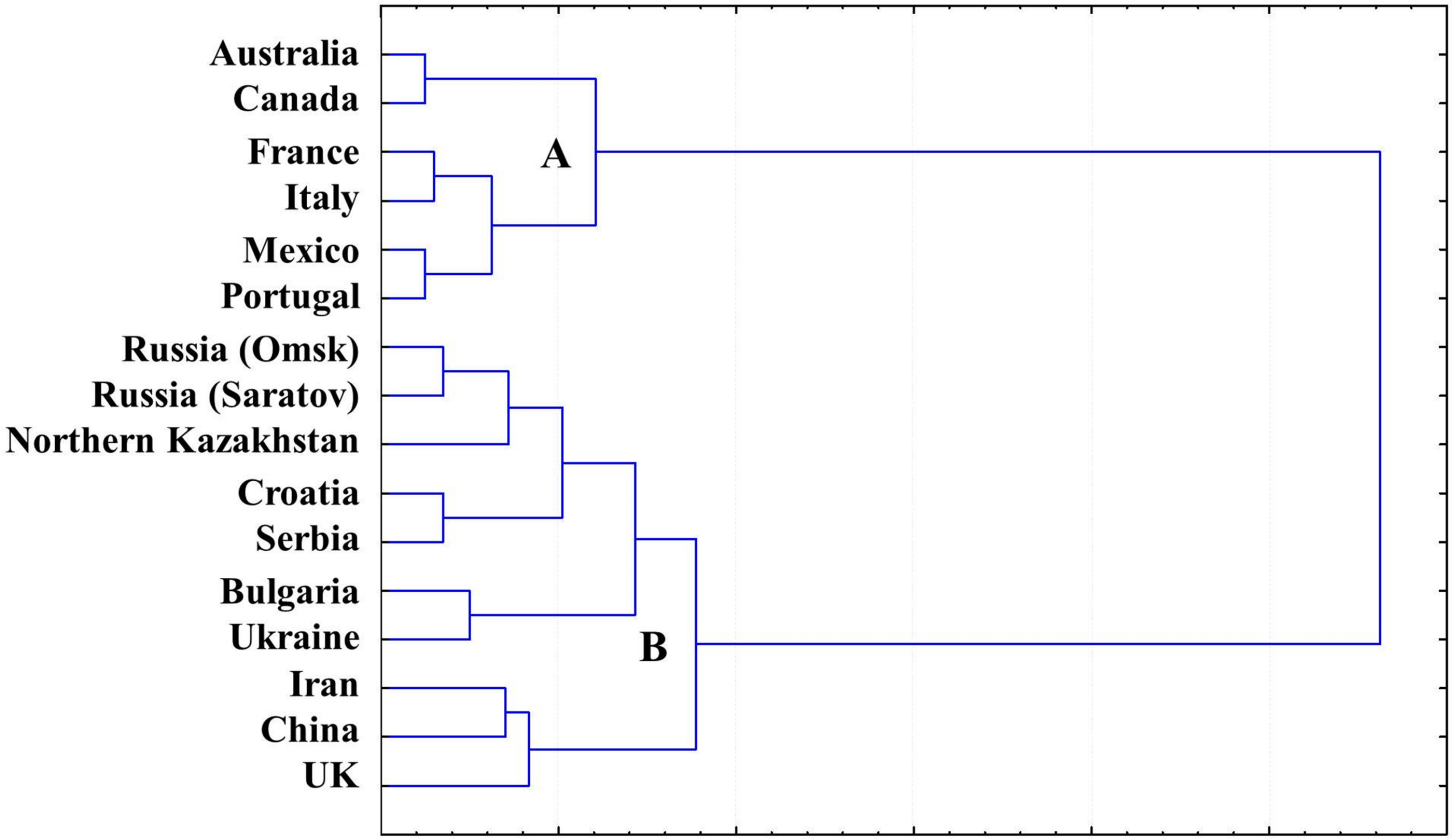
Gliadin dendrogram showing the allele diversity in *Gli* loci of bread wheat from Northern Kazakhstan and other countries.

A gliadin dendrogram (Fig. 4) was established based on a cluster analysis of our combined current and previously published results of allele variation in the *Gli* loci and GGF, in wheat originating from Northern Kazakhstan (Supplemental Information 1 and 2) and other publicly available data for wheat from other countries (Table 2).

**Table 2:** GGF of wheat cultivars from various countries compared to the GGF of Northern Kazakhstan wheats.

Countries/regions	Gliadin coding loci *Gli*						References
	*A1*	*B1*	*D1*	*A2*	*B2*	*D2*	
Australia	***g***	***b***	***f***	***c***	***c***	***w***	*Metakovsky et al. (2018)*
Canada	***m***	***d***	*j* + *a*	***m***	***c***	*h* + *m*	
France	*af* + *c*	*b* + *f*	***b***	***m***	*o* + *c*	***h***	
Italy	***a***	***g***	***k***	*g* + *o*	***o***	***a***	
Mexico	*o* + *a*	*d* + *b*	*b* + *a*	***f***	***c***	*m* + *j*	
Portugal	***a***	*c* + *l*	***b***	***f***	***c***	*c* + *j*	
Russia (Saratov)	***f***	***e***	***a***	***q***	***s***	***e***	
Bulgaria	*b* + *a*	***b***	***b***	*b* + *g*	***b***	***b***	
Croatia	*a* + *b*	***e***	***a***	***e***	***e***	*m* + *a*	
Serbia	*a* + *b*	*b* + *l*	***b***	*b* + *g*	***b***	*b* + *a*	
Ukraine	***b***	***b***	***g***	*f* + *b*	***b***	***e***	
Russia (Omsk)	***f***	***e***	***a***	***q***	***b***	***a***	Novoselskaya-Dragovich, Fisenko & Puhalskii (2013)
Iran	***f***	***f***	***b***	*g* + *l*	***o***	*a* + *n* + *h*	*Salavati et al. (2008)*
China	*o* + *a*	*l* + *e*	*a* + *f*	*g* + *f*	*I* + *h*	*b* + *a*	*Novoselskaya-Dragovich et al. (2011)*
UK	***f***	***f***	***b***	***l***	***g***	***a***	*Chernakov & Metakovsky (1994)*
Northern Kazakhstan	***f***	***e***	***a***	***q***	***t***	***q***	Current study

Two major Clades (designated as A and B) were found in GGF, with strong separation of the analysed accessions. Wheat genotypes from Australia, America and Western Europe form Clade A, while the more diverse Clade B includes accessions with *Gli* alleles mostly distributed in Eastern Europe and Asia, with the exception of the UK. As expected, all wheat cultivars from Northern Kazakhstan had GGF most closely related to Russian wheats, particularly those developed in the two big Breeding Research Institutes in Saratov and Omsk, in the European and Siberian part of Russia, respectively. These regions are very close to Northern Kazakhstan geographically (Fig. 1), and also have a long history of exchange of wheat germplasms within the former Soviet Union.

## Discussion

The presented study is an important part of the breeding program for seed quality in wheat, to illustrate breeder selections for wheat genotypes with various combinations of gliadin alleles. The received results can be used as the basis of a breeding strategy for wheat genotype selection with preferred GGF and favourable combinations of *Gli* alleles. In the current study, genetic origin, gliadin characteristics and the value of breeding for alleles in each of gliadin-coding loci in 70 wheat accessions will be discussed in separate sub-sections.

### Locus *Gli-A1*

Fifteen *Gli-A1* alleles were identified in the current study in wheat cultivars from Northern Kazakhstan, out of 29 alleles published in the recent Catalogue of gliadin-coding genes (Metakovsky et al., 2018). The highest frequency (0.39) was found in genotypes with allele ***f***. The wide-spread occurrence of the allele ***f*** in 27 wheat accessions from Northern Kazakhstan out of the 70 studied seems to be related to introgression of the following high grain quality cultivars: Cesium 111 (***f***), Albidum 24 (***f***) and Saratovskaya 29 (***j*** + ***f***) in the early stages of the wheat breeding process in Kazakhstan (Metakovsky et al., 2006).

The possible origin and spread of other *Gli-A1* alleles, ***i***, ***o*** and ***b***, is likely also related to wheat cultivars from Russia. For example, a series of cultivars entitled Omskaya 20, 22 and 23, originating from the forest-steppe zone of South-Western Siberia, with allele *Gli-A1**o***, seems to be used in the exchange breeding process (Metakovsky et al., 2006). This conclusion is similar to those in our previous published results using a different set of wheat cultivars from Northern Kazakhstan (Utebayev et al., 2016).

### Locus *Gli-B1*

Very limited genetic diversity was found in the *Gli-B1* locus, where the single allele ***e*** showed the absolute highest frequency at 62% (Fig. 3). It is important to note that this allele is quite widely distributed, especially in southern regions of European Russia (Novoselskaya-Dragovich et al., 2003) as well as in South-Eastern and South-Western Siberia, which are close and directly neighbouring to Northern Kazakhstan, respectively (Nikolaev, Pukhal’sky & Upelniek, 2009). The occurrence and quite frequent distribution of the allele *Gli-B1**e*** may be directly related to the actively-used popular Russian drought tolerant cultivars with elite grain quality from the Saratov region: Albidum 43 (***f,*** ***e******, a, q, o, a***), Lutescence 62 (***j,*** ***e******, a, q, o, a***), and Saratovskaya 29 (***j***+***f,*** ***e******, a, q*** + ***s, q*** + ***s, e***) (Metakovsky et al., 2006). However, these cultivars had some disadvantages, particularly a sensitivity to a range of diseases (Morgounov, Rosseeva & Koyshibayev, 2007).

The second allele, *Gli-B1**b,*** with three-fold less frequency (29%) has a much wider distribution among wheat cultivars from Scandinavian countries to Australia (Metakovsky et al., 2018) and may therefore indicate the wide adaptability of genotypes with this allele. It is very likely that the *Gli-B1**b*** allele is originated from historic and classical winter wheat cultivars bred in the former Soviet Union, Besostaya 1 (***b,*** ***b******, b, b, b, b***) and Mironovskaya 808 (***f,*** ***b******, g, n, m, e***) (Metakovsky et al., 2006).

### Locus *Gli-D1*

There were eight alleles identified in this locus in the studied wheat accessions, which is exactly half of all that were published in the recent Catalogue of gliadin-coding loci (Metakovsky et al., 2018). Three alleles showed the highest range of frequencies: *Gli-D1**a***, 0.34; *-****b***, 0.31; and *-****f***, 0.16 (Fig. 3). However, the spectrum of genetic diversity in the present study slightly differed from our paper published earlier with another set of bread wheat accessions from Northern Kazakhstan, where only four *Gli-D1* alleles were identified with the following frequencies: allele ***a***, 44.2%; and each of alleles ***f*** and ***i***, 23.3%, respectively (Utebayev et al., 2016).

Similar to those indicated for other alleles above, Russian wheat cultivars were widely used in the initial breeding programs in Northern Kazakhstan. Therefore, it is very likely that the most commonly distributed allele, ***a***, is originated from one or several cultivars, particularly Albidum 43, Lutescence 62, or Saratovskaya 29 (Chernakov & Metakovsky, 1994; Nikolaev, Pukhal’sky & Upelniek, 2009). Additionally, this allele, *Gli-D1**a***, had quite high frequencies among wheat cultivars in Southern Kazakhstan, with a very different environment, but the origin of the allele ***a*** from the former Soviet Union wheat germplasm genepool is not in doubt (Absattarova, 2002). This statement is in complete consensus with data for GGF in Kazakh wheats published in a recent review (Metakovsky et al., 2018). The comparison of world-wide distribution of *Gli* allele ***a*** among wheat genotypes bred and grown in Croatia, Finland and Spain (Sontag-Strohm, 1997; Metakovsky et al., 2018), indicated for a possible association between allele *Gli-D1* ***a*** with adaptability of wheat plants to various environments.

It is important to note that two *Gli-D1* alleles, ***a*** and ***f***, encode the synthesis of almost identical spectra of gliadin components. The only additional gliadin component present with smaller size in the *γ*-zone of protein electrophoresis was recorded in wheat genotypes *Gli-D1* with allele ***a*** but not with allele ***f***. Therefore, it is hypothesised that wheat genotypes *Gli-D1**a*** and *-****f*** can have very similar gliadin gene nucleotide sequences (Chebotar et al., 2012).

The moderately distributed allele ***b*** is also very likely to have originated from foreign wheat accessions introgressed earlier in the Kazakh breeding program. However, it is interesting that the *Gli-D1**b*** allele originates from a very different genepool of winter wheat, rather than spring wheat. This statement is based on published data showing a quite high distribution of the allele ***b*** among winter wheat, but not in spring wheat, in the former Soviet Union (Kozub et al., 2009; Novoselskaya-Dragovich et al., 2015). Therefore, we can speculate that the possible introgression of the *Gli-D1**b*** allele from winter wheat can indicate for the wide adaptability of wheat genotypes, regardless of their responses to cold and vernalisation.

### Locus *Gli-A2*

The *Gli-2* gene is much more diverse in wheat, where the smallest number of alleles were recorded in *Gli-A2* and accounted for 14 (Fig. 3) of the 39 registered in the recent Catalogue of *Gli* alleles (Metakovsky et al., 2018). The most commonly distributed alleles among the studied wheat cultivars from Northern Kazakhstan were: *Gli-A2**b*** (17.1%), *-****f*** (12.1%), and *-****q*** (15.0%). The first allele ***b*** was very typical for wheat cultivars from very diverse geographical regions and had similarities to wheats from the UK, Eastern Europe and the Krasnodar region in the southern part of Russia (Metakovsky et al., 2018). Winter wheat germplasm accessions also had about 22% of the allele *Gli-A2**b*** (Novoselskaya-Dragovich et al., 2015), and this allele is particularly spread among wheat cultivars with high tolerance to cold temperatures (Markarova, 2015). This leads us to the conclusion that the *Gli-A2**b*** allele may be associated with genotypes with high adaptability to unfavourable conditions for plant growth.

The allele *Gli-A2**f*** was present in wheat cultivars originating from the Saratov region, Russia (Novoselskaya-Dragovich, Fisenko & Puhalskii, 2013) and in some winter wheat cultivars (Novoselskaya-Dragovich et al., 2015) but is known to show the highest frequencies in spring wheat from Mexico and Portugal (Metakovsky et al., 2018).

The third allele, *Gli-A2**q,*** was very likely introgressed and spread widely in wheat cultivars in Northern Kazakshtan from germplasm originating from the nearby Russian regions of Saratov and Omsk (Novoselskaya-Dragovich, Fisenko & Puhalskii, 2013). For example, cv. Lutescence 62 was widely used for hybridisations in Kazakhstan with GGF (***j, e, a,*** ***q******,*** ***o, a***) from the Saratov Breeding Institute, and it was consequently bred during individual selection of plants of the original historical cv. Poltavka (***f*** + ***j, e, a,*** ***q***+ ***k, o, a*** + ***e***) (Rutz, 2005; Metakovsky et al., 2006). The influence of the wheat genepool originating from the Saratov region on the wheat breeding program in Northern Kazakhstan was described in the genetic polymorphism of *Gli* alleles in papers published a relatively long time ago (Sozinov, Metakovsky & Koval, 1986; Metakovsky et al., 1988). However, among Kazakh wheat cultivars with elite quality of grain, only the allele *Gli-A2**q*** had the highest frequency of distribution, indicating for a possible genetic association with high grain quality (Dobrotvorskaya et al., 2009).

### Locus *Gli-B2*

Seventeen out of 45 *Gli* alleles described in recent Catalogues (Metakovsky et al., 2018) were identified and analysed in the current study. The highest frequency was found for the allele *Gli-B2**t***, 12.8%, followed by 10.7% for alleles *-****b*** and *-****g***, respectively. The origin of the first allele ***t*** remains unclear because it was registered as a minor *Gli* allele in some modern wheat cultivars from the Omsk Breeding Station, Russia (Chernakov & Metakovsky, 1994). We can propose that the origin of the allele *Gli-B2**t*** is likely related to the old Russian cv. Cesium 111 used for hybridisations with GGF (***f, m, i, j,*** ***t******, i***) and published earlier (Metakovsky et al., 2006; Morgounov, Rosseeva & Koyshibayev, 2007).

The occurrence and distribution of allele *Gli-B2**b*** is definitely related to the use and introgression of wheat accessions from Eastern Europe and Russia, where this allele was exclusively present (Metakovsky et al., 2018). In contrast, the *Gli* allele ***g*** very likely originates from one of the wide geographically dispersed countries such as the Scandinavian group (Metakovsky et al., 2018), the UK (Chernakov & Metakovsky, 1994), France (Metakovsky & Branlard, 1998), and China (Novoselskaya-Dragovich et al., 2011).

### Locus *Gli-D2*

The sixth and last gliadin-coding locus, *Gli-D2*, was present with 14 alleles. The three most widely distributed alleles were ***q***, ***b*** and ***a***, with corresponding percentage of frequencies: 23.5%, 17.8% and 11.4%, respectively. In the comparison with gliadin allele distributions, *Gli-D2**b*** was originated from Russian wheat germplasm (Metakovsky et al., 2018). Both ***q*** and ***a*** alleles were widely distributed in local wheats from Northern Kazakhstan, and regarding our previous study, allele *Gli-D2**a*** was for the first time found in three Kazakh wheat cultivars, Milturum 45, Tzelinogradka and Snegurka (Utebayev et al., 2016). These three cultivars were included in wheat breeding in Northern Kazakhstan as genetic donors, and the first two of them (Milturum 45 and Tzelinogradka) were bred from original, old and polymorphic cv. Cesium 111 with GGF—***f, m, i, j, t,*** ***a***+ ***e*** (Metakovsky et al., 2006). It is more likely that modern Kazakh wheat genotypes with allele *Gli-D2**a*** had a pedigree progenitor from one of the biotypes of cv. Cesium 111. Less likely, but still possible, is that the origin of the ***a*** allele is from other countries where it was found, such as Croatia, Germany, France, Holland, Italy, Scandinavian countries, Spain or the UK (Metakovsky et al., 2018), indicating for a possible wide interest for wheat breeding programs.

### Comparison of genetic diversity between *Gli-1* and *Gli-2* alleles

In both our current and previous study (Utebayev et al., 2016), the three most popular and widely distributed modern spring bread wheat cultivars from Northern Kazakhstan with elite grain quality have the following GGF: Akmola 2 (***g, e, a, i, e, s***), Astana (***g*** + ***j, e, f*** + ***i, p, h, b***), and Karabalykskaya 90 (***i*** + ***m*** + ***f, e, a*** + ***g, q*** + ***l, v, a***) (Supplemental Information 2). These cultivars have a similar composition of alleles in the gene *Gli-1,* with three homeologous loci (*-A1*, *-B1* and *-D1*) to wheat cultivars with very high grain quality from the Russian Breeding Institutes, Saratov and Omsk. Therefore, it was hypothesised that allele compositions in each of three loci of *Gli-1* were directly related to grain and baked bread quality and its improvement (Li et al., 2009; Novoselskaya-Dragovich, Fisenko & Puhalskii, 2013). In contrast, allele compositions in the second gene *Gli-2* with three homeologous loci (*-A2*, *-B2* and *-D2*) located in chromosome group 6, were genetically associated with possible adaptation of plants to a dry environment (Novoselskaya-Dragovich, Fisenko & Puhalskii, 2013).

Such a conclusion, made from the comparison between *Gli-1* and *Gli-2* genes, may explain how non-pedigree related wheat cultivars from various geographic regions with a different climate have very similar or identical compositions of *Gli-1* alleles. This is because one of the main targets of wheat breeding is the production of wheat with elite quality of grain and baked bread, where genetic diversity for allele composition in *Gli-1* is much smaller than in *Gli-2*: 14, 6 and 8 alleles for *Gli-A1*, *-B1* and *-D1*; and 14, 17 and 14 alleles for *Gli-A2*, *-B2* and *-D2*, respectively (Fig. 3). It is possible that a single perfect pedigree genotype with excellent grain quality was used as a progenitor in many modern wheat cultivars, providing limited genetic variability in allele composition of *Gli-1*. In contrast, the *Gli-2* gene, with much wider variability in allele compositions, was more likely involved in plant adaptation to a dry environment. Because such environments are quite variable in different countries and geographic regions, it may be reflected in and explain the higher variability in allele diversity in *Gli-2*. The presented results reflect the efforts of wheat breeders over many years of artificial selection based on phenotyping variability in grain quality and tolerance to dry environments, as apparent in the results of genetic diversity in both gliadin-coding genes based on gliadin analyses.

## Conclusions

Genetic diversity in the alleles of gliadin-coding genes *Gli-1* and *Gli-2* was studied, and gliadin genetic formulas were established following the results of gliadin electrophoresis in a set of 70 spring bread wheat cultivars from Northern Kazakhstan. The *Gli* alleles with highest frequencies in the studied wheat material were identified as follows: *Gli-A1**f*** (39.3%), *-B1**e*** (71.9%), *-D1**a*** (41.0%), *-A2**q*** (17.8%), *-B2**t*** (13.5%), and *-D2**q*** (20.4%). This allele combination of both *Gli* genes was the most widely distributed in Northern Kazakhstan, and genotypes with such gliadin formula can be used as prospective breeding material for elite grain quality and better adaptability to the dry environment of the Northern Kazakhstan region and for wheat breeding under similar conditions.

##  Supplemental Information

10.7717/peerj.7082/supp-1 Supplemental Information 1 Gliadin genetic formulas (Current study) Gliadin genetic formulas of bread wheat from Northern Kazakhstan (Current study).

10.7717/peerj.7082/supp-2 Supplemental Information 2 Gliadin genetic formulas (Previous studies) Gliadin genetic formulas of bread wheat from Northern Kazakhstan (Previous studies).

## Additional Information and Declarations

### Data Availability

The following information was supplied regarding data availability: The raw data are available in the Supplemental Files.

## References

[ref-1] Absattarova AS (2002). Identification of Kazakhstan agroecotypes winter common wheat cultivars using of gliadin components blocks. Dissertation of the candidate of biological sciences.

[ref-2] Aguiriano E, Ruiz M, Fité R, Carrillo JM (2008). Genetic variation for glutenin and gliadins associated with quality in durum wheat (*Triticum turgidum* L. ssp. *turgidum*) landraces from Spain. Spanish Journal of Agricultural Research.

[ref-3] Al-Doss AA, Al-Hazmi AS, Dawabah AAM, Abdel-Mawgood AA, Al-Rehiayani SM, Solaiman A, Moustafa K, Motawei M (2010). Impact of Cre and peroxidase genes of selected new wheat lines on cereal cyst nematode (*Heterodera avenae* Woll) resistance. Australian Journal of Crop Science.

[ref-4] Borisjuk N, Kishchenko O, Eliby S, Schramm C, Anderson P, Jatayev S, Kurishbayev A, Shavrukov Y (2019). Genetic modification for wheat improvement: from transgenesis to genome editing. BioMed Research International.

[ref-5] Burridge AJ, Wilkinson PA, Winfield MO, Barker GL, Allen AM, Coghill JA, Waterfall C, Edwards KJ (2018). Conversion of array-based single nucleotide polymorphic markers for use in targeted genotyping by sequencing in hexaploid wheat (*Triticum aestivum*). Plant Biotechnology Journal.

[ref-6] Bushuk W, Zillman RR (1978). Wheat cultivar identification by gliadin electrophoregrams. I. Apparatus, method and nomenclature. Canadian Journal of Plant Science.

[ref-7] Chebotar SV, Blagodarova EM, Kurakina EA, Semenyuk IV, Polishchuk AM, Kozub NA, Sozinov IA, Khokhlov AN, Ribalka AI, Sivolap YuM (2012). Genetic polymorphism of loci determining bread making quality in Ukrainian wheat varieties. Vavilov Journal of Genetics and Breeding.

[ref-8] Chernakov VM, Metakovsky EV (1994). Diversity of gliadin-coding locus allelic variants and evaluation of genetic similarity of common wheat varieties from different breeding counters. Genetika.

[ref-9] Dobrotvorskaya TV, Dragovich AY, Martynov SP, Pukhal’skii VA (2009). Genealogical and statistical analyses of the inheritance of gliadin-coding alleles in a model set of common wheat *Triticum aestivum* L. cultivars. Russian Journal of Genetics.

[ref-10] Ghanti SK, Sujata KG, Rao S, Udayakumar M, Kishor PBK (2009). Role of enzymes and identification of stage-specific proteins in developing somatic embryos of chickpea (*Cicer arietinum* L.). In Vitro Cellular and Developmental Biology—Plant.

[ref-11] Hailegiorgis D, Lee CA, Yun SJ (2017). Allelic variation at the gliadin coding loci of improved Ethiopian durum wheat varieties. Journal of Crop Science and Biotechnology.

[ref-12] Henry RJ, Furtado A, Rangan P (2018). Wheat seed transcriptome reveals genes controlling key traits for human preference and crop adaptation. Current Opinion in Plant Biology.

[ref-13] (1993).

[ref-14] Jatayev S, Kurishbaev A, Zotova L, Khasanova G, Serikbay D, Zhubatkanov A, Botayeva M, Zhumalin A, Turbekova A, Soole K, Langridge P, Shavrukov Y (2017). Advantages of Amplifluor-like SNP markers over KASP in plant genotyping. BMC Plant Biology.

[ref-15] Khan K, Hamada AS, Patek J (1985). Polyacrylamide gel electrophoresis for wheat variety identification: effect of variables on gel properties. Cereal Chemistry.

[ref-16] Khlestkina EK, Shumny VK (2016). Prospects for application of breakthrough technologies in breeding: the CRISPR/Cas9 system for plant genome editing. Russian Journal of Genetics.

[ref-17] Kozub NA, Sozinov IA, Sobko TA, Kolyuchii VT, Kuptsov SV, Sozinov AA (2009). Variation at storage protein loci in winter common wheat cultivars of the central forest-steppe of Ukraine. Cytology and Genetics.

[ref-18] Kusznir J (2015). Russia’s borders: Moscow’s long alliance with Kazakhstan is strong but not unbreakable. https://theconversation.com/russias-borders-moscows-long-alliance-with-kazakhstan-is-strong-but-not-unbreakable-36457.

[ref-19] Li Y, Song Y, Zhou R, Branlard G, Jia J (2009). Detection of QTLs for bread-making quality in wheat using a recombinant inbred line population. Plant Breeding.

[ref-20] Liang Z, Chen K, Zhang Y, Liu J, Yin K, Qiu JL, Gao C (2018). Genome editing of bread wheat using biolistic delivery of CRISPR/Cas9 *in vitro* transcripts or ribonucleoproteins. Nature Protocols.

[ref-21] Markarova ZhR (2015). Frost hardiness and yield of soft winter wheat cultivars and accessions in the Rostov region. Nauchny Zhurnal Rossiiskogo NII Problem Melioratsii.

[ref-22] McIntosh RA, Yamazaki Y, Devos KM, Dubkovsky J, Rogers J, Appels R (2008). MacGene 2003. Catalogue of gene symbols for wheat. http://wheat.pw.usda.gov/ggpages/wgc/2003.

[ref-23] Melnikova NV, Ganeva GD, Popova ZG, Landjeva SP, Kudryavtsev AM (2010). Gliadins of Bulgarian durum wheat (*Triticum durum* Desf.) landraces: genetic diversity and geographical distribution. Genetic Resources and Crop Evolution.

[ref-24] Melnikova NV, Kudryavtseva AV, Kudryavtsev AM (2012). Catalogue of alleles of gliadin-coding loci in durum wheat (*Triticum durum* Desf.). Biochimie.

[ref-25] Metakovsky EV (1991). Gliadin allele identification in common wheat. 2 Catalogue of gliadin alleles in common wheat. Journal of Genetics and Breeding.

[ref-26] Metakovsky EV, Branlard G (1998). Genetic diversity of French common wheat germplasm based on gliadin alleles. Theoretical and Applied Genetics.

[ref-27] Metakovsky EV, Branlard GP, Graybosch RA, Wrigley C, Bekes F, Bushuk W (2006). Gliadins of common wheat: polymorphism and genetics. Gliadin and glutenin: the unique balance of wheat quality.

[ref-28] Metakovsky EV, Branlard G, Graybosch RA, Bekes F, Cavanagh CR, Wrigley CW, Bushuk W (2006). The gluten composition of wheat varieties and genotypes. Part I. Gliadin composition table. https://www.aaccnet.org/initiatives/definitions/Documents/GlutenFree/I_Gliadin.pdf.

[ref-29] Metakovsky EV, Koval SF, Movchan VK, Sozinov AA (1988). Genetic formulas of gliadin in cultivars of common wheat of Northern Kazakhstan. Selekciya i Semenovodstvo.

[ref-30] Metakovsky E, Melnik V, Quijano MR, Upelniek V, Carrillo JM (2018). A catalog of gliadin alleles: polymorphism of 20th-century common wheat germplasms. The Crop Journal.

[ref-31] Metakovsky EV, Novoselskaya AY (1991). Gliadin allele identification in common wheat. 1. Methodological aspects of the analysis of gliadin pattern by one-dimensional polyacrylamide—gel electrophoresis. Journal of Genetics and Breeding.

[ref-32] Morgounov A, Rosseeva L, Koyshibayev M (2007). Leaf rust of spring wheat in Northern Kazakhstan and Siberia: incidence, virulence, and breeding for resistance. Australian Journal of Agricultural Research.

[ref-33] Nei M (1973). Analysis of gene diversity in subdivided populations. Proceedings of the National Academy of Sciences of the United States of America.

[ref-34] Netsvetaev VP, Akinshina OV, Bondarenko LS (2010). Genetic control of several *α*-amylase isozymes in winter hexaploid wheat. Russian Journal of Genetics.

[ref-35] Nikolaev AA, Pukhal’sky VA, Upelniek VP (2009). Genetic diversity of local spring bread wheats (*Triticum aestivum* L.) of West and East Siberia in gliadin genes. Russian Journal of Genetics.

[ref-36] Nirmal RC, Furtado A, Rangan P, Henry RJ (2017). Fasciclin-like arabinogalactan protein gene expression is associated with yield of flour in the milling of wheat. Scientific Reports.

[ref-37] Nirmal RC, Furtado A, Wrigley C, Henry RJ (2016). Influence of gene expression on hardness in wheat. PLOS ONE.

[ref-38] Novoselskaya-Dragovich AY, Bespalova LA, Shishkina AA, Melnik VA, Upelniek VP, Fisenko AV, Dedova LV, Kudryavtsev AM (2015). Genetic diversity of common wheat varieties at the gliadin-coding loci. Russian Journal of Genetics.

[ref-39] Novoselskaya-Dragovich AY, Fisenko AV, Puhalskii VA (2013). Genetic differentiation of common wheat cultivars using multiple alleles of gliadin coding loci. Russian Journal of Genetics.

[ref-40] Novoselskaya-Dragovich AY, Fisenko AV, Yankovsky NK, Kudryavtsev AM, Yang Q, Lu Z, Wang D (2011). Genetic diversity of storage protein genes in common wheat (*Triticum aestivum* L.) cultivars from China and its comparison with genetic diversity of cultivars from other countries. Genetic Resources and Crop Evolution.

[ref-41] Novoselskaya-Dragovich AY, Krupnov VA, Saifulin RA, Pukhalskiy VA (2003). Dynamics of genetic variation at gliadin-coding loci in Saratov cultivars of common wheat *Triticum aestivum* L. over eight decades of scientific breeding. Russian Journal of Genetics.

[ref-42] Obukhova LV, Shumny VK (2016). The inheritance of endosperm storage proteins by the line of the Saratovskaya 29 cultivar of common wheat from its parental forms. Russian Journal of Genetics.

[ref-43] Palosuo K, Varjonen E, Kekki OM, Klemola T, Kalkkinen N, Alenius H, Reunala T (2001). Wheat *ω*-5 gliadin is a major allergen in children with immediate allergy to ingested wheat. The Journal of Allergy and Clinical Immunology.

[ref-44] Pastorello EA, Farioli L, Conti A, Pravettoni V, Bonomi S, Iametti S, Fortunato D, Scibilia J, Bindslev-Jensen C, Ballmer-Weber B, Robino AM, Ortolani C (2007). Wheat IgE-mediated food allergy in European patients: *α*-amylase inhibitors, lipid transfer proteins and low-molecular-weight glutenins. International Archives of Allergy and Immunology.

[ref-45] Qi PF, Wei YM, Yue YW, Yan ZH, Zheng YL (2006). Biochemical and molecular characterization of gliadins. Molecular Biology.

[ref-46] Rutz RI (2005). Breeding center of siberian research institute of agriculture is a leader of Siberian breeding. Vestnik VOGiS.

[ref-47] Salavati A, Sameri H, Boushehri AAS, Yazdi-Samadi B (2008). Evaluation of genetic diversity in Iranian landrace wheat *Triticum aestivum* L. by using gliadin alleles. Asian Journal Plant Science.

[ref-48] Sánchez-León S, Gil-Humanes J, Ozuna CV, Giménez MJ, Sousa C, Voytas DF, Barro F (2018). Low-gluten, non-transgenic wheat engineered with CRISPR/Cas9. Plant Biotechnology Journal.

[ref-49] Scheben A, Batley J, Edwards D (2017). Genotyping-by-sequencing approaches to characterize crop genomes: choosing the right tool for the right application. Plant Biotechnology Journal.

[ref-50] Shavrukov Y (2016). Comparison of SNP and CAPS markers application in genetic research in wheat and barley. BMC Plant Biology.

[ref-51] Shewry PR, Halford NG (2001). Cereal seed storage proteins: structures, properties and role in grain utilization. Journal of Experimental Botany.

[ref-52] Sontag-Strohm T (1997). Gliadin and glutenin subunit alleles on group 1 chromosomes in Finnish spring wheats. Acta Agriculturae Scandinavica, Section B—Soil and Plant Science.

[ref-53] Sozinov AA, Metakovsky EV, Koval SF (1986). Patterns of genotype formation in the breeding of the wheat. Vestnik Selskohozyaistvennoi Nauki.

[ref-54] Sozinov AA, Poperelya FA (1980). Genetic classification of prolamins and its use for plant breeding. Annales de Technologie Agricole.

[ref-55] Tkachuk R, Metlish VJ (1980). Wheat cultivar identification by high voltage gel electrophoresis. Annales de Technologie Agricole.

[ref-56] Utebayev M, Dashkevich S, Babkenov A, Shtefan G, Fahrudenova I, Bayahmetova S, Sharipova B, Kaskarbayev Zh, Shavrukov Y (2016). Application of gliadin polymorphism for pedigree analysis in common wheat (*Triticum aestivum* L.) from Northern Kazakhstan. Acta Physiologiae Plantarum.

[ref-57] Xynias IN, Kozub NO, Sozinov IA (2006). Seed storage protein composition of Hellenic bread wheat cultivars. Plant Breeding.

[ref-58] Zhang Y, Li D, Zhang D, Zhao X, Cao X, Dong L, Liu J, Chen K, Zhang H, Gao C, Wang D (2018). Analysis of the functions of *TaGW2* homoeologs in wheat grain weight and protein content traits. The Plant Journal.

[ref-59] Zhivotovsky LA (1980). An index intrapopulation diversity. Zhurnal Obshchei Biologii.

[ref-60] Zhivotovsky LA (1991). Population Biometrics.

