# Supplementary material for: Genetic diversity of gliadin-coding alleles in bread wheat (Triticum aestivum L.) from Northern Kazakhstan
Source: PeerJ. 2019 Jun 12;7:e7082. doi: 10.7717/peerj.7082 (PMC6571009; doi:10.7717/peerj.7082)
Supplement: Supplemental Information 1 — Gliadin genetic formulas of bread wheat from Northern Kazakhstan (Current study). [file peerj-07-7082-s001.pdf]

Supplementary material 1

Gliadin genetic formulas of bread wheat from Northern Kazakhstan (Current study)

Cultivars/advanced lines	Gliadin-coding loci (*Gli*)					
	A1	*B1*	*D1*	*A2*	*B2*	*D2*
Karabalyk Agricultural Breeding Station						
Aina	***a***	***e***	***b***	***k***	***o***	***l***
Avgustina	***i***	***b***	***a***	***f***	***l***	***f***
Bostandyk	***m***	***b+e***	***a***	***l***	***r***	***s***
Byrlestyk	***m***	***b***	***c***	***k***	***l***	***h***
Erythrospermum 35-12-13	***f***	***e***	***f***	***c***	***g***	***a***
Erythrospermum 59	***k***	***b***	***b+h***	***f***	***o***	***m***
Erythrospermum 78	***f***	***b***	***a***	***n***	***v***	***p***
Galateya	***f***	***e***	***g***	***d***	***r***	***a***
K - 36544	***h***	***e***	***d***	***f***	***i***	***j***
K - 51122	***h***	***b***	***b***	***n***	***n***	***j***
Karabalykskaya 20	***f***	***b***	***b***	***l***	***t***	***b***
Karabalykskaya 38	***m+j+f***	***e***	***a***	***d***	***j***	***e***
Karabalykskaya 4	***f***	***e***	***g***	***l***	***a***	***n***
Karabalykskaya 7	***b***	***b***	***f+a***	***b***	***f***	***a***
Karabalykskaya 8	***f***	***b***	***a***	***a***	***o***	***q***
Karabalykskaya 9	***o+f***	***b+e***	***a***	***a+d***	***c+g***	***m+q***
Karabalykskaya 98	***o***	***e***	***a***	***n***	***o***	***a***
Komsomol'skaya 29	***k***	***e***	***f***	***s***	***b***	***b***
Komsomol'skaya 90	***i+m+f***	***e***	***a+g***	***q+l***	***v***	***a***
Korneevka	***f***	***l***	***h***	***f***	***k***	***n***
Line 22 ChS	***r***	***e***	***h***	***d***	***t***	***l***
Line 4-10-16	***f***	***e***	***g***	***m***	***t***	***b***
Line S 19ChS	***f***	***l***	***f***	***f***	***e***	***b***
Lutescens 12	***f***	***b***	***i+b***	***s***	***b***	***e***
Lutescens 13	***b***	***e+b***	***b***	***b***	***t***	***l***
Lutescens 2	***c***	***e+b***	***b***	***p***	***r***	***a***
Lutescens 20	***b***	***e***	***b***	***g***	***b***	***b***
Lutescens 22	***c***	***e+b***	***b***	***p***	***r***	***a***
Lutescens 26	***p***	***e***	***b***	***l+f***	***g***	***q***
Lutescens 3	***f***	***e***	***c***	***k***	***g***	***e***
Lutescens 33	***i***	***e***	***a***	***b***	***l+i***	***m+c***
Lutescens 36	***g***	***e***	***b***	***p***	***r***	***q***
Lutescens 41	***c***	***b***	***f***	***s***	***r***	***k***
Lutescens 48-204-03	***f***	***e***	***b***	***b***	***t***	***l***

Lutescens 54	*q*	*e*	*g*	*l*	*m*	*q*
Simkar 20	*h*	*e*	*b*	*k*	*o*	*m*
Tomiris	*o*	*e*	*f*	*q*	*g*	*m*
Tumar	*i*	*e*	*a*	*f*	*f*	*f*
Zhana-Kyzyl	*o*	*g*	*f*	*k*	*t*	*p*
Zhazira	*i*	*e*	*b*	*b*	*n*	*s*
Pavlodar science research institute of agriculture						
1266-87-13-94-23	*f*	*m*	*a*	*n*	*m*	*e*
22,90-94-4	*f*	*e+b*	*f*	*n*	*b*	*q*
26,89-94-49	*m*	*e*	*g*	*b*	*g*	*q*
31,85-94-80	*o*	*b*	*a*	*q*	*m*	*q*
35,86-94-166	*f*	*e*	*a*	*f*	*m*	*b+h*
48,87-94-3	*r*	*e*	*a*	*q*	*m*	*q*
7,89-64-16	*f*	*e*	*a*	*b*	*a*	*q*
Lutescens 12/93-01-4	*i*	*e*	*a*	*q*	*r*	*q*
Lutescens 1266-87-13-94-23	*f*	*k*	*a*	*q*	*g*	*q*
Lutescens 16/93-01-08	*i*	*e*	*a*	*b*	*d*	*n*
Lutescens 17-89-94-17	*o*	*b*	*b*	*n*	*n*	*q*
Lutescens 2.86-94-64	*f*	*k*	*f*	*f*	*v*	*s*
Lutescens 24.90-94-1	*b*	*e*	*b*	*b*	*b*	*q*
Lutescens 24-90-94-2	*f*	*e*	*h*	*h*	*t*	*b*
Lutescens 25/93-01-2	*f*	*e*	*a*	*q*	*a*	*l*
Lutescens 261	*f*	*e*	*f+a*	*q+m*	*b+l*	*l+m*
Lutescens 29-94	*h*	*e*	*b*	*d*	*f*	*b*
Lutescens 30	*i*	*b*	*b*	*b*	*a*	*b*
Lutescens 30.89-94-11	*f*	*e+b*	*f*	*n*	*b*	*q*
Lutescens 30-94	*c*	*b*	*b*	*l*	*t*	*b*
Lutescens 35-86-94-166	*b*	*e*	*b*	*b*	*b*	*q*
Lutescens 53-95	*k*	*b*	*b*	*l*	*n*	*m*
Lutescens 65	*f*	*e*	*h+b*	*h+q*	*f+o*	*s+l*
Lutescens 86-91-94-1	*f*	*e*	*f*	*k*	*t*	*b*
Lutescens 9-33	*f*	*b*	*h*	*q*	*l*	*a*
Pavlodarskaya 10	*i*	*e*	*a*	*l*	*a*	*q*
Pavlodarskaya 11	*f*	*e*	*a*	*d*	*d*	*n*
Pavlodarskaya 9	*k*	*e*	*a*	*q*	*g*	*l*
Pavlodarskaya 93	*i*	*b*	*b*	*b*	*a*	*b*
Pyrotrix 35-86	*r*	*e*	*a*	*q*	*e*	*b*
