# Supplementary material for: Genetic diversity of gliadin-coding alleles in bread wheat (Triticum aestivum L.) from Northern Kazakhstan
Source: PeerJ. 2019 Jun 12;7:e7082. doi: 10.7717/peerj.7082 (PMC6571009; doi:10.7717/peerj.7082)
Supplement: Supplemental Information 2 — Gliadin genetic formulas of bread wheat from Northern Kazakhstan (Previous studies). [file peerj-07-7082-s002.pdf]

Supplementary material 2

Gliadin genetic formulas of bread wheat from Northern Kazakhstan (Previous studies)

Cultivars/advanced lines	Gliadin-coding loci (*Gli*)						Reference
	A1	*B1*	*D1*	*A2*	*B2*	*D2*	
Oral	i	e	a	b	o	q	Utebayev et al., 2018a
Soltystyk	h	e	a	l	o+d	q+s	
Tauelsyzdyk 20	f	e	a	q	m	q	
Shortandinskaya 2014	f	e	a	l	g	q	
Shortandinskaya 2015	f	e	f	q	t	q	
Karabalykskaya 90	f	e	f	c	r	e	Mrzabek et al., 2017
Karabalykskaya 91	f	e	f	c	m	q	
Karabalykskaya 92	o	e	a	f	r	i	
Komsomolskaya 3	o	e	a	m	g	q	
Komsomolskaya 18	o	e	l	f	f	p	
Lutescens 4	f	e	b	t	r	q	
Lutescens 14	m	e	a	n	l	m	
Lutescens 28	o	e	b	w	t	a	
Lutescens 52	g	f	a	c	c	m	
Lutescens 71	f	l	a	b	l	m	
Albidum 113119	f+o	e	a+f	g	t+f	q	Utebayev et al., 2018b
Albidum P 3119	i	e	a	q	o	m	
Erythrospermum 2945	f	e	a	q	f	m	
Gornostepnaya	f	e	f	q	g	p	
Ishimskaya 100	m	b	f	r	r	d	
K–4541	m	f	j	k	e	b	
K–36677	f	m	f	k	t	l	
K–37146	j	e	a	q	o	i	
Lutescens 45	f+h	e	a	q+n+f	t+v+c+a	q+a+l	
Priirtyshskaya 86	m+n	b	a	l+s	g+l	s+b	
Uralskaya yubileynaya	f	e	f	q	g	g	
Akmola 2	g	e	a	i	e	s	Utebayev et al., 2016
Akmola 3	f+o	e	f	p	g	a	
Akmola 40	o	e	a	p	d	e	
Akmolinka 1	q	m	g	k	d	s	
Astana	g+j	e	f+i	p	h	b	
Astana 2	o+f	e+h	f	p	l	e	
Asyl-Sapa	h	e	i	g	c	l	
Baiterek	o	e	i	s	c	d	
Dostyk	i	e	a	s	s	n	
Ishimskaya 88	f	f	f	b+p	d	e	
Ishimskaya 90	o	e	a	s	h	c	
Ishimskaya 92	g	e	f	p	l	c	
Ishimskaya 98	f	e	i+f	b+p	v	b	

Kenzhegali	***f***	***e***	***i+f***	***k+p***	***t***	***a+l***
Lastochka	***q***	***e***	***g***	***k***	***d***	***e***
Lutescens 38	***p***	***e***	***i***	***q***	***s***	***s***
Lutescens 94	***f+d***	***e***	***i+f***	***c+p***	***d***	***e***
Lutescens 268	***f***	***e***	***a***	***l***	***g***	***i***
Milturum 45	***f***	***m***	***i***	***k***	***t***	***a***
Pamyati Movchana	***f***	***e***	***a***	***q***	***s***	***a***
Pyrotrix 28	***p***	***m***	***g***	***k***	***d***	***a***
Shortandinka	***p***	***m***	***g***	***k***	***o***	***s***
Shortandinskaya yubileynaya	***f***	***e***	***a***	***l***	***f***	***q***
Shortandinskaya 25	***i***	***e***	***a***	***c***	***s***	***s***
Shortandinskaya 95 uluchsh.	***y***	***e***	***f***	***l***	***t***	***a***
Shortandinskaya 125	***c***	***e***	***f***	***p***	***a***	***c***
Shortandinskaya 2007	***i***	***e***	***a***	***q***	***e***	***a***
Shortandinskaya 2012	***f***	***e***	***a***	***i***	***t***	***p***
Snegurka	***f***	***e+j***	***a***	***k***	***d***	***a***
Tselina 50	***o***	***e***	***i***	***n***	***q***	***e***
Tselinnaya yubileynaya	***f***	***e***	***a***	***q***	***d***	***e***
Tselinnaya 3S	***i***	***e***	***a***	***p***	***s***	***k***
Tselinnaya 20	***f***	***e***	***a***	***q***	***d***	***e***
Tselinnaya 21	***f***	***e***	***a***	***q***	***d***	***e***
Tselinnaya 24	***g***	***e***	***a***	***s***	***s***	***s***
Tselinnaya 26	***i+c***	***e***	***a***	***c***	***s***	***s***
Tselinnaya 60	***i***	***e***	***a***	***c***	***s***	***s***
Tselinnaya 90	***f***	***e***	***i***	***p***	***e***	***b***
Tselinnaya 2007	***o***	***e***	***f***	***q***	***g***	***q***
Tselinnaya 2008	***q***	***e***	***a***	***l***	***t***	***q***
Tselinogradka	***f***	***e***	***i***	***k***	***t***	***a***
Vladimir	***g***	***e***	***f***	***p***	***l***	***c***

Mrzabek AA, Utebayev MU, Sharipova BU. 2017. Identification of bread wheat (*Triticum aestivum* L.) by polymorphism of gliadin-coding loci in Northern Kazakhstan. In: *Actual Problems of Science and Education in the Field of Natural and Agricultural sciences. Scientific and Practical Conference*. Petropavlovsk, Kazakhstan, 17 February 2017, 130-131 [In Russian].

Utebayev M, Dashkevich S, Babkenov A, Shtefan G, Fahrudenova I, Bayahmetova S, Sharipova B, Kaskarbayev Z, Shavrukov Y. 2016. Application of gliadin polymorphism for pedigree analysis in common wheat (*Triticum aestivum* L.) from Northern Kazakhstan. *Acta Physiologiae Plantarum* **38(8):**204 DOI 10.1007/s11738-016-2209-4

Utebayev MU, Bome NA, Dashkevich SM. 2018a. Identification alleles of gliadin-coding loci in varieties of bread wheat from A.I. Barayev RPC of Grain Farming. In: *Tobolsk Nauchnyi - 2018, Scientific and Practical Conference*. Tobolsk, Russia, 15-16 November 2018, 88-90 [In Russian].

Utebayev MU, Dashkevich SM, Bome NA, Chilimova IV, Kradetskaya OO. 2018b. Identification and registration common wheat on electrophoretic spectrum of the seed storage proteins. In: *Science and Innovation:Vectors of the Development. International Scientific and Practical Conference of Young Scientists*. Barnaul, Russia, 24-25 October 2018. Vol. 1, 54-56 [In Russian].
